# Differing Dietary Nutrients and Diet-Associated Bacteria Has Limited Impact on Spider Gut Microbiota Composition

**DOI:** 10.3390/microorganisms9112358

**Published:** 2021-11-15

**Authors:** Wang Zhang, Fengjie Liu, Yang Zhu, Runhua Han, Letian Xu, Jie Liu

**Affiliations:** 1 Hubei Key Laboratory of Regional Development and Environmental Response, Faculty of Resources and Environmental Science, Hubei University, Wuhan 430062, China; zhangxiaowang@stu.hubu.edu.cn; 2 The State Key Laboratory of Biocatalysis and Enzyme Engineering of China, College of Life Sciences, Hubei University, Wuhan 430062, China; liufengjie0819@163.com (F.L.); zhuyang316@163.com (Y.Z.); 3 McKetta Department of Chemical Engineering, University of Texas at Austin, Austin, TX 78712, USA; runhuahan@utexas.edu; 4 School of Nuclear Technology and Chemistry, Biology University of Science and Technology, Xianning 437100, China

**Keywords:** gut microbiota, spider, diet regime, ants, nutrient, diet-associated microbes

## Abstract

Spiders are a key predator of insects across ecosystems and possess great potential as pest control agents. Unfortunately, it is difficult to artificially cultivate multiple generations of most spider species. Since gut bacterial flora has been shown to significantly alter nutrient availability, it is plausible that the spiders’ microbial community plays a key role in their unsuccessful breeding. However, both the gut microbial composition and its influencing factors in many spiders remain a mystery. In this study, the gut microbiota of *Campanicola campanulata*, specialists who prey on ants and are widely distributed across China, was characterized. After, the impact of diet and diet-associated bacteria on gut bacterial composition was evaluated. First, two species of prey ants (*Lasius niger* and *Tetramorium caespitum*) were collected from different locations and fed to *C. campanulata.* For each diet, we then profiled the nutritional content of the ants, as well as the bacterial communities of both the ants and spiders. Results showed that the protein and carbohydrate content varied between the two prey ant species. We isolated 682 genera from 356 families in the ants (dominant genera including *Pseudomonas*, *Acinetobacter*, *Paraburkholderia*, *Staphylococcus,* and *Novosphingobium*), and 456 genera from 258 families in the spiders (dominated by *Pseudomonas*). However, no significant differences were found in the gut microbiota of spiders that were fed the differing ants. Together, these results indicate that nutritional variation and diet-associated bacterial differences have a limited impact on the microbial composition of spider guts, highlighting that spiders may have a potentially stable internal environment and lay the foundation for future investigations into gut microbiota.

## 1. Introduction

Spiders are some of the most successful animals that use venom for their survival and arguably consume the most diverse range of prey [1]. They play a crucial role in biological pest control in paddy fields, orchards, cotton fields, and tea gardens [2,3,4]. Consequently, the artificial protection and breeding of spiders in both agricultural and forest systems can provide an alternative pest management strategy to chemical insecticides while also facilitating ecological restoration. However, artificially breeding most spiders has proven challenging, either due to external environmental controls (temperature, humidity, etc.) or inner factors resulting from the environment determining the female sexual maturity, inducing the low spider survival rates in the laboratory [5]. Previous studies have shown that while spiders can digest a variety of foods, several spider taxa are specialized to prey on ants [6], which are also difficult to breed artificially. According to previous studies, bacteria associated with spider diets could be involved in the unsuccessful breeding of host animals (e.g., Pollock et al. 2017 demonstrated that diet is linked to breeding success in blue tits (*Cyanistes caeruleus*) [7]). Together with the difference that most of the spiders are omnivorous with active predation. Thus, we speculate that the specialized mono-dietary regimes contribute to the low survival rates of the spiders in a lab setting. Whether the unsuccessful spider breeding attempts are linked to a nutrient deficiency and how these dietary regimens impact spiders remain unknown.

Some evidence has suggested that the gut bacterial community has a significant impact on the host’s digestion, nutritional acquisition, and juvenile development [8], which are often linked to the overall community structure [9,10,11]. Previous studies have examined both gut bacterial diversity and biological functions in a wide range of arthropods [12,13,14], demonstrating the critical role of the bacterial community found in arthropod guts [15,16,17]. Previous studies have also illustrated that the composition of the gut microbiota is easily influenced by numerous biotic and abiotic factors, including gut structure, the physiological environment, pH, oxygen levels, host immune system, and the developmental stage. Of these, diet and dietary microbiota are the most important factors [18,19]. Considering their complicated dietary regimens and diverse gut microbiota, spiders provide a suitable model to explore the degree to which diet or diet-acquired microbes impact the gut bacterial community. Many studies on microorganisms within spiders have focused on endosymbionts and their reproductive effects on their hosts [20,21,22], while other studies have identified and compared the gut microbiomes in different spider species [23,24,25,26]; analyzed the heritability of microbial communities within spiders [27]; and demonstrated that the gut microbiota of the spider *Ariamnes waikula* (Araneae, Theridiidae), which is found in the Hawaiian islands, to be fairly conserved for over 2 million years [28]. Although the existence of a common core microbiome in spiders remains elusive, gut bacteria in genera *Acinetobacter*, *Pseudomonas*, *Bacillus* are dominant and widely distributed in more than 40 spiders [23,24,25], while gut bacteria *Staphylococcus*, *Corynebacterium*, and *Cutibacterium* have varied abundance among the spiders. However, considering both the abundance and variety of spiders, there is a lack of knowledge about their gut microbiota and how this bacterial composition is influenced or maintained [7].

*Campanicola campanulata* [29] is widely distributed across China and plays an important role in the soil arthropod community [30]. While most spiders are euryphagous, it is one of the few spiders to predate ants, making it a unique case study. *C. campanulata* lives in a bell-shaped nest that opens downward. It is made up of small, dry branches and fallen leaves and is normally found in sand or clay near the soil layer. This can effectively deceive predators and increase their chances of survival. In this study, two ants preyed upon by *C. campanulata* (*Lasiu niger* and *Tetramorium caespitum*) were collected from different geographic sites and fed to the spiders. The nutritional content in both ants was then quantified, as were the 16S rRNA V3–V4 high variable regions of the feed ants and the gut bacterial community of *C. campanulata*. Despite huge dietary variation at both the nutritional and microbial levels, our study highlighted a potentially stable internal environment in spiders and laid the foundation for future investigations into spider gut microbiota.

## 2. Materials and Methods

### 2.1. Sample Collection and Rearing

*C. campanulata* were collected from a field in Yingshan, Hubei, China (31°5′33″ N, 115°48′26″ E). All samples were collected during the same season from April to June 2019, transported to the laboratory, and starved for at least 7 days before the feed experiments. The ants selected as the food source for this study were divided into three groups according to their species and collection sites: *Lasius niger* (LN_WH) and *Tetramorium caespitum* (TC_WH) were collected from Hubei University, Wuhan, China (30°24′39.09″ N, 114°19′56.88″ E), and another group of *Tetramorium caespitum* (TC_LY) was collected from the Shuanglong mountain forest park, Luoyang, Henan, China (34°31′4.32″ N, 112°43′53.06″ E). The spiders were then randomly divided into three groups and fed on one ant type (CC-LN_WH, CC-TC_WH, CC-TC_LY); a total of 3–5 ants were put into the container at a time, and the ant carcasses were checked to confirm that spiders had fed. To exclude the potential influence of ants’ food on the microbiome of ants, all field captured ants were put into the containers to feed the spiders directly. Each group of spiders was fed with only one type of ants from the beginning to the end. Spiders were dissected 48 h after feeding. The spiders and ants used in this study were all identified as non-endangered and non-protected species by the first author (families Theridiidae and Formicidae). Authors are required to confirm whether aspects of their research abide by ethical principles proclaimed by Hubei University.

Before dissection, spiders were surface sterilized with 70% ethanol and rinsed three times with sterile water to remove surface contaminants [7,26,31,32]. While this wash will not entirely remove surface contamination, it should not enable a high signal in gut microbiota analysis. The oral cavity and stomach were separated, and the entire abdomen was excised for testing. The midguts were not removed due to their small sizes. Gut samples were stored in 1.5 mL microcentrifuge tubes at −80 °C for further analysis (six spider guts were pooled together into one sample, *n* = 5). Ant analysis was performed directly on the whole body (ten ants were pooled together into one sample, *n* = 5). Ants were washed with 70% ethanol and sterile water and kept at −80 °C until DNA extraction. Three groups of spiders (CC-LN_WH, CC-TC_WH, CC-TC_LY) and three groups of ants (LN_WH, TC_WH, TC_LY) were collected. All operations were performed in an aseptic environment.

### 2.2. Measurements of Dietary Nutrients in Ants

Proteins, carbohydrates, and lipids are primary structural units in ants and the main nutritional constituents in the diet of Arthropoda. Four nutritional indices were quantified in the feed ants, including protein, glucose, triglyceride (TG), and trehalose concentrations. Before quantification, each ant sample was weighted with a precision electronic microbalance (METTLER TOLEDO). Considering that each individual ant was very small, fifteen ants were combined into one sample for analyzed concurrently (*n* = 6). The ants were then pulverized in liquid nitrogen using a Tissuelyser-24 (Jingxin Limited Company, Shanghai, China). The protein content of each sample was analyzed with an Easy II protein quantitative kit (Beijing TranGen Biotech Co., Ltd., Beijing, China). The optical density at 562 nm (OD_562_) of the purple compound after 30 min of biuret reaction (60 °C) was assayed using a micro-plate reader (SpectraMax plus384, Molecular Devices, San Jose, CA, USA). Glucose content in the ants was determined using a glucose assay kit (Shanghai Rongsheng Biotech Co., Ltd., Shanghai, China), following the manufacturer’s protocol. After incubating the ant samples with the reagents in the kit for 10 min at 37 °C, the OD_505_ value of the solution was qualified using the micro-plate reader. Similarly, the triglyceride (TG) and trehalose content in each sample was determined using a Triglyceride Assay kit (OD at 510 nm, 10 min of incubation at 37 °C, Nanjing Jiancheng Bioengineering Institute, Nanjing, China) or a trehalose assay kit (OD at 620 nm, 10 min of incubation at 95 °C, Nanjing Jiancheng Bioengineering Institute, Nanjing, China) following the manufacturer’s instructions. All four kits contained standard samples to standardize the final results.

### 2.3. DNA Extraction and PCR Amplification

Microbial community genomic DNA of each pooled sample was extracted using a DNeasy Blood and Tissue Kit (Qiagen, Hilden, Germany) following the manufacturer’s instructions. The DNA extract was checked on 1% agarose gel, and DNA concentration and purity were determined with NanoDrop 2000 UV-vis spectrophotometer (Thermo Scientific, Wilmington, CA, USA). The hypervariable region V3-V4 of the bacterial 16S rRNA gene were amplified with primer pairs (338F: 5′-ACTCCTACGGGAGGCAGCAG-3′ and 806R: 5′-GGACTACHVGGGTWTCTAAT-3′) [33] by an ABI GeneAmp^®^ 9700 PCR thermocycler (ABI, Vernon, CA, USA), with an eight-base sequence barcode unique to each sample at the 5′ end of 338F and 806R, respectively. The PCR amplification was carried out in a total volume of 20 μL containing 0.8 μL of each primer, 10 ng of template DNA, 4 μL of 5× FastPfu Buffer, 2 μL of 2.5 mM dNTPs, 0.4 μL of FastPfu Polymerase, 0.2 μL of BSA, and Supplement ddH_2_O to 20 μL. The following parameters were used in the PCRs: denaturation for 3 min at 95 °C and 30 cycles of denaturation for 30 s at 95 °C, annealing for 30 s at 53 °C, and elongation at 72 °C for 45 s. For the last cycle, the elongation time was extended to 10 min at 72 °C. The PCR product was extracted from 2% agarose gel and purified using the AxyPrep DNA Gel Extraction Kit (Axygen Biosciences, Union City, CA, USA) according to manufacturer’s instructions and quantified using QuantiFluor™-ST (Promega, Madison, WI, USA).

### 2.4. Illumina MiSeq Sequencing and Bioinformatic Processing

Total community DNA was used for amplification and sequencing of the 16S rRNA, targeting the variable V3–V4 regions. Purified amplicons were pooled in equimolar and paired-end sequenced on an Illumina MiSeq platform (Illumina, San Diego, CA, USA) according to the standard protocols by Majorbio Bio-Pharm Technology Co., Ltd. (Shanghai, China). The raw 16S rRNA gene sequencing reads were demultiplexed, quality-filtered by Trimmomatic, and merged by FLASH with the following criteria: (i) the 300 bp reads were truncated at any site receiving an average quality score of <20 over a 50 bp sliding window, and the truncated reads shorter than 50 bp were discarded; (ii) exact barcode matching, 2 nucleotides mismatch in primer matching, and reads containing ambiguous characters were removed; and (iii) only overlapping sequences longer than 10 bp were assembled according to their overlapped sequence. Reads that could not be assembled were discarded. Operational taxonomic units (OTUs) with 97% that often used in QIIME (1.9.1) similarity cutoff were clustered using UPARSE (version 7.1, http://drive5.com/uparse/, accessed on 27 July 2021), and chimeric sequences were identified and removed using UCHIME. The taxonomy of each OTU representative sequence was analyzed by RDP Classifier (http://rdp.cme.msu.edu/, accessed on 27 July 2021) against the 16S rRNA database using a confidence threshold of 0.7. All steps were implemented in a Snakemake workflow. The raw pyrosequencing data were obtained and deposited in the NCBI Sequence Read Archive under the BioProjects ID: PRJNA742878.

### 2.5. Statistical Analyses

The experimental design consisted of six groups, including three kinds of ants (LN_WH, TC_WH, TC_LY) and the spiders (CC-LN_WH, CC-TC_WH, CC-TC_LY) eating different ants. For downstream analysis, we took the obtained OTU table and prepared a “filtered table” (v.2.5) using QIIME (1.9.1) custom scripts. First, we extracted from the OTUs table the bacteria domain using the command split_otu_table_by_taxonomy.py. OTUs tables were rarefied to a minimum sample depth, while rarefaction curves were produced with the vegan package in R and evaluated whether the sequencing depth was saturated. To calculate the diversity indexes, we used the alpha_diversity.py and alpha_rarefaction.py commands to obtain Shannon, Simpson, ACE, and Chao1, observed OTU. A Venn diagram was visualized using the R package software to show unique and shared microbial compositions. We compared the diversity indices between spider or ant groups using a one-way analysis of variance (ANOVA). ANOVA was also used to assess the statistical differences in genus abundance between the spider or ant groups. Results with *p* < 0.05 between groups were considered statistically significant. Principal coordinate analysis (PCoA) of the Bray–Curtis dissimilarity distances were conducted at the genus level, and a permutational multivariate analysis of variance, based on the weighted UniFrac distance (PERMANOVA), was used to identify differences in genus abundance between the sample groups using the QIIME software package. A heatmap was generated based on the relative abundance of the genera. Data were analyzed using SPSS 19.0, and figures were drawn using GraphPad Prism 6.

## 3. Results

### 3.1. Analysis of Nutritional Composition

**Figure 1:**
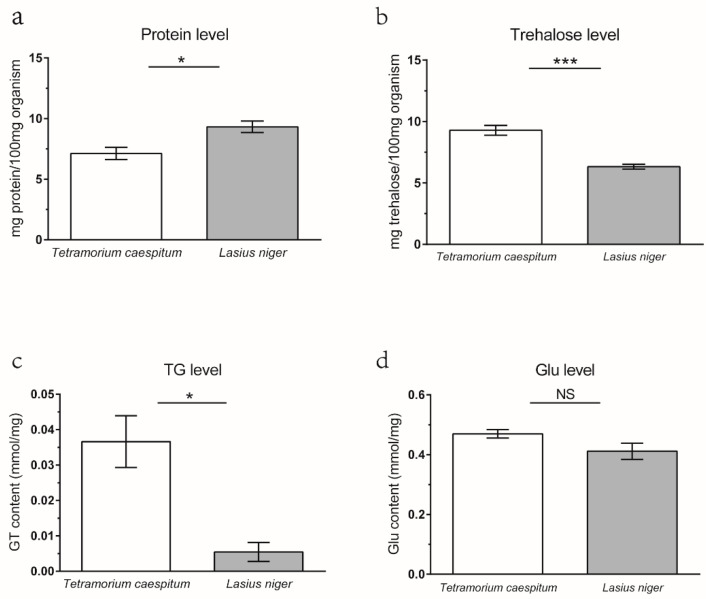
The content of protein (**a**), trehalose (**b**), TG (triglyceride) (**c**), and glucose (**d**) in *Tetramorium caespitum* and *Lasius niger*. Asterisks indicate statistically significant differences (* *p* < 0.05; *** *p* < 0.001); NS, not significant. Each measurement contains six to eight replicates.

We quantified the nutritional composition of three different varieties of ants, revealing significant differences between *L. niger* and *T. caespitum* species regardless of their collection sites (Figure 1), while no difference was found for the same species of ants collected from two different sites (Figure S1). The concentration of TG in *L. niger* (0.0055 mmol/mg organism) was markedly lower than in *T. caespitum* (0.0314 mmol/mg, *p* < 0.05; Figure 1c). Similarly, trehalose levels in *L. niger* (6.322 mg/100 mg organism) were significantly lower than in *T. caespitum* (9.284 mg/100 mg organism, *p* < 0.001; Figure 1b). Conversely, the protein concentration in *L. niger* (0.093 mg/100 mg organism) was distinctly higher than *T. caespitum* (0.071 mg/100 mg organism, *p* < 0.05; Figure 1a). Lastly, there were no significant differences in glucose content between the two ant species (0.319–0.411 mmol/mg for both; Figure 1d) or between ants of the same species from different locations. These data highlighted the substantial nutritional difference between the two prey ant species.

### 3.2. The Diversity Analysis of the Bacterial Community

In total, 1,677,627 usable sequences were obtained from 29 samples of spiders. Among which, 1,116,358 high-quality sequences were selected, with an average of 38,495 sequences per sample. A total of 258 genera were detected in the *C. campanulata* combining three groups of statistics of the spiders. Again, at the genus level, the sequences could be assigned to 682 genera. The number of each sample was listed in Table S1. Table S2 lists genera with an abundance of at least 1% of the total number of OTUs found in the sample. The rarefaction curves were constructed based on S_obs_ to verify the adequacy of the sampling depth and were generated from randomly subsampled data sets with the same number of 16S sequences. The rarefaction curve reached saturation for each sequencing sample (Figure S2). The rank-abundance curve showed that the OTUs of all samples increased gradually and then held at stable values with increasing numbers of measured sequences, indicating that most bacterial sequences obtained by the MiSeq sequencing system reflect the abundance and diversity of the microbiota (Figure S3). The CC-LN_WH curve had a large range on the horizontal axis, meaning that the richness of this group was higher than the other two groups. The bacterial species in the CC-TC_LN group was more evenly distributed because its curve was flatter than in the other two groups (Figure S3).

### 3.3. Composition of Bacterial Community

**Figure 2:**
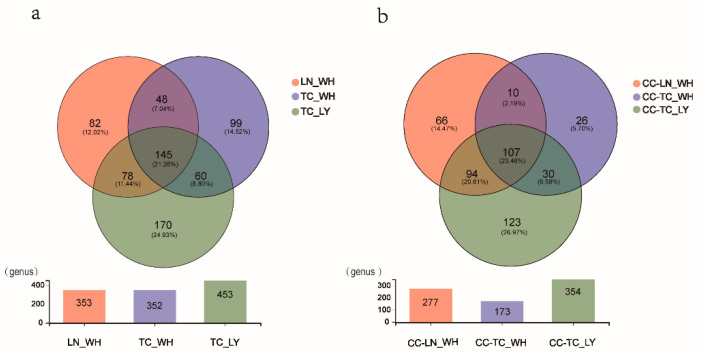
Venn diagram demonstrating the quantities of common and unique microorganism species at genus level between (**a**) ant groups and (**b**) spider groups. TC_LY, *T. caespitum* Luoyang; TC_WH, *T. caespitum* Wuhan; LN_WH, *L. niger* Wuhan; CC-, spiders fed different diets. Each measurement contains six replicates.

To facilitate the analysis of which microbial species were common or unique among the samples, while Venn diagrams of the different groups were drawn based on their source: spiders or ants at the genus level (Figure 2). The gut microbial composition among spiders showed no significant differences; however, the quantity of microbial OTUs in spiders fed with *T. caespitum* from Wuhan was significantly lower than the other groups, even though there were not fewer *T. caespitum* species than in other ants. The alpha diversity was estimated using the ACE, Chao1, Shannon, and Simpson indices (Table 1), while there were no significant differences between the gut samples of *C. campanulata* for the OTU numbers or the ACE, Chao1, Shannon, and Simpson indices. However, the Shannon index of the TC_LY group was significantly higher value, and the Simpson’s index was significantly lower than the other two groups of ants (Table 1, Duncan’s new multiple range test, *p* < 0.01).

**Figure 3:**
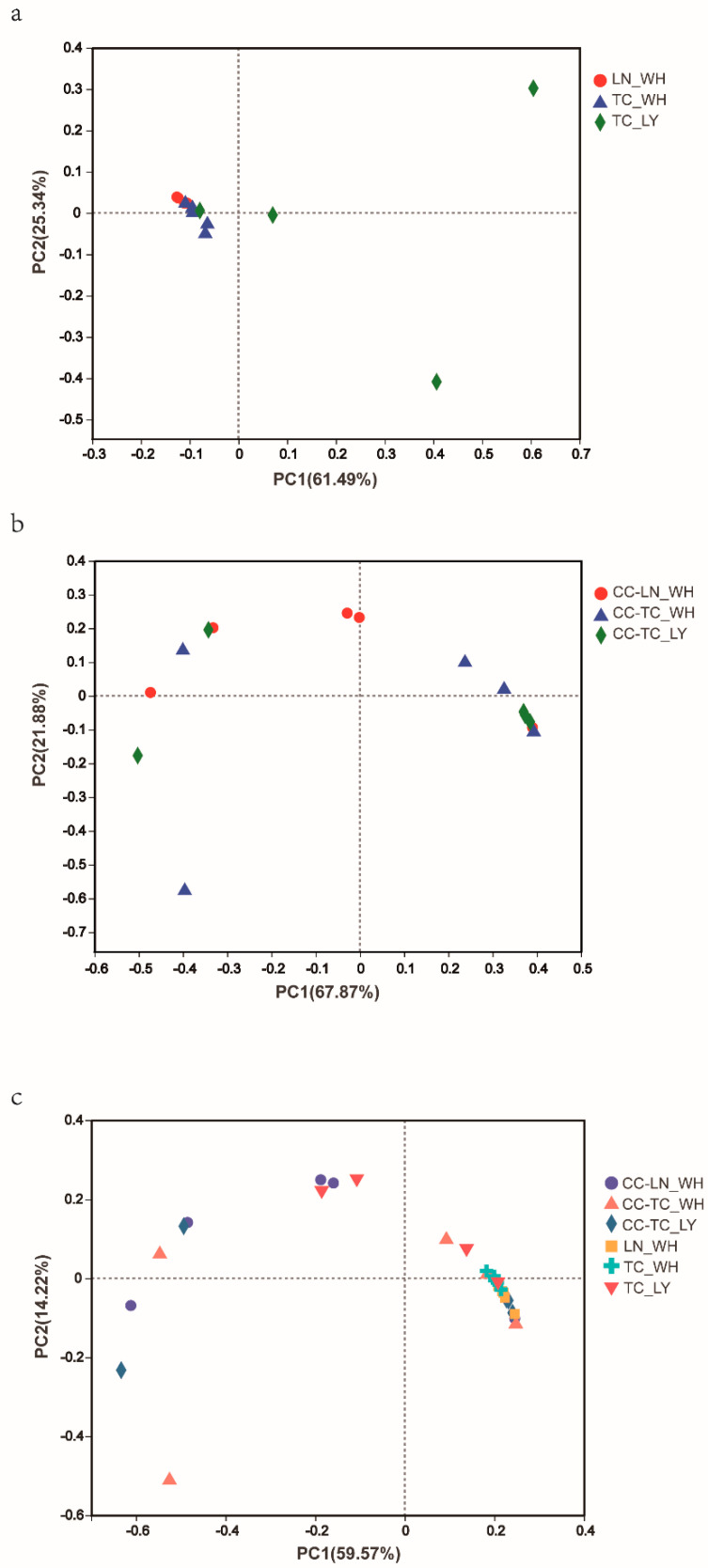
Principal coordinate analysis (PCoA) plots of the bacterial community for ants (**a**) and spiders (**b**) and together (**c**) based on Bray−Curtis distance matrix (PERMANOVA *p* < 0.05). Each symbol represents one sample. TC_LY, *T. caespitum* Luoyang; TC_WH, *T. caespitum* Wuhan; LN_WH, *L. niger* Wuhan; CC−, spiders fed different diets.

**Figure 4:**
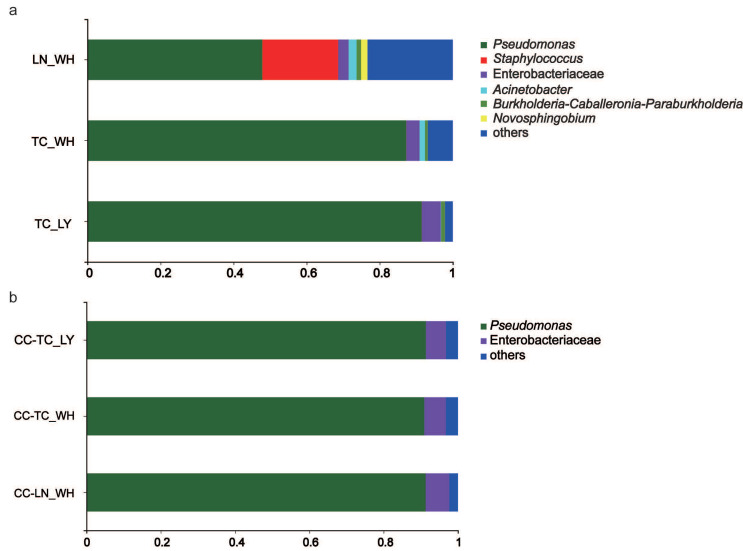
Taxonomic composition of bacterial community at the genus level in ants (**a**) and spiders (**b**). TC_LY, *T. caespitum* Luoyang; TC_WH, *T. caespitum* Wuhan; LN_WH, *L. niger* Wuhan; CC-, spiders fed different diets.

PCoA of the weighted UniFrac distance resulted in a 2-dimensional solution of ants in which PC1 accounted for 61.49% of the variation and PC2 for 25.34% (Figure 3a, PERMANOVAR, *p* = 0.002). PCoA based on the spiders revealed that the first two principal component scores accounted for 67.87% and 21.88% of the variation, respectively (Figure 3b, PERMANOVAR, *p* = 0.776) and together with the last one (Figure 3c) These graphs demonstrate that all three spider groups were clustered together, indicating that their communities have a similar composition. The ant groups differed significantly (Table S3). Bar graphs exhibiting the differences in the six dominant species of microbiota further validate the findings of PCoA (Figure 4). While there was significant variation among ants (Figure 4a), the dominant microbiota species in spiders were of the same genus (Figure 4b). The genus with the highest relative abundance was *Pseudomonas*.

**Figure 5:**
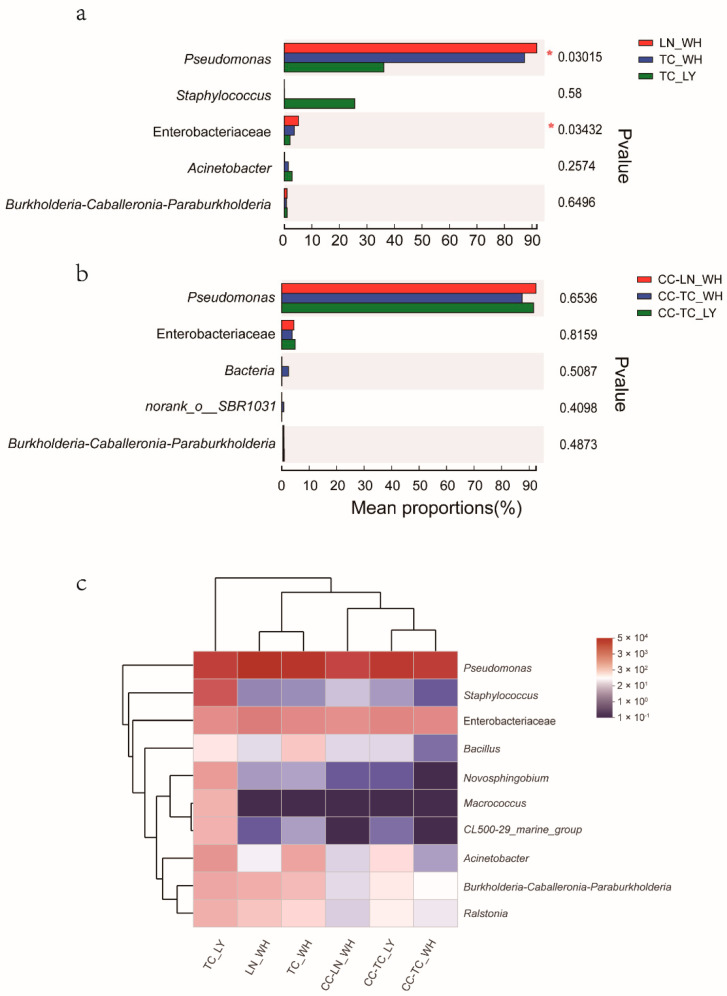
Comparison of the top five most abundant genera in ants (**a**) and spiders (**b**). *, *p* ≤ 0.05. Heatmap (**c**) shows the relative abundances of the ten most abundant genera in the six sample groups. Dendrograms for hierarchical cluster analysis grouping genera and sample locations are shown at the left and top, respectively. The color scale represents the normalized values of relative abundances by log10. Zero values were added as 1 and log10 transformed. TC_LY, *T. caespitum* Luoyang; TC_WH, *T. caespitum* Wuhan; LN_WH, *L. niger* Wuhan; CC-, spiders fed different diets.

Quantified comparison of the five most abundant genera revealed no significant differences in the relative abundances of microbiota, at the genus level, among the three spider groups (Figure 5b). The composition of ant microbiotas distinguished *T. caespitum* from Luoyang as significantly less abundant than ants from Wuhan in both *Pseudomonas* and Enterobacteriaceae (Figure 5a). A heat map, in which the difference in the abundance distribution of species between samples could be quantified by distance graphically, showed that at genus, the microbial community in the six groups of spiders or ants. It also revealed that there were differences in the abundance of the microbial community phyla in some different groups (Figure 5c). *Pseudomonas* had the highest abundance among the six groups and had different relative abundances in both spiders and ants. *T. caespitum* from Luoyang also differed from the other two ant-feed groups (Figure 5c). Overall, these results indicate that there was no clear division among the three groups of spiders but that the ants could not be clustered into a single region.

## 4. Discussion

It is becoming increasingly understood that the gut microbiota can influence body development, physiology, and ecological inter- or intraspecific interactions [34,35,36,37]. The microbial community, however, is also plastic. Several factors can alter the composition of the gut microbial community [18,19] and thus have the potential to shape the community function [18,38]. According to prior studies, the influence of many factors on the microbiota is context-dependent and might only be observed above a certain threshold; for instance, the composition of the predominant termite gut microbiota remained largely constant despite variations in diet [39]. In contrast, dietary differences markedly altered the gut bacterial community in gypsy moths [40]. However, studies on whether and/or which factors shape the gut microbiota of widespread predators, such as spiders, that play a key role in ecosystem stability have rarely been reported. We fed spiders with three groups of ants, which all had different nutritional contents and microbiota. We then profiled the gut bacterial communities of the spiders under different dietary conditions in an attempt to determine how variations in nutrients or diet-associated bacteria affected spiders’ gut microbiota but did not reach perfect conclusions because many factors varied with the different prey ants. Considering the relatively slow digestion and the striking endurance of starvation of the spider, the spiders were dissected 48 h after feeding with ants. Furthermore, a pilot experiment showed that starvation for at least 7 days had no obvious effect on the total abundance of the spider’s gut microbiota. Our results demonstrated that the gut bacterial communities were similar across diets, despite differences in the nutritional content of ingested microbes. Discrepancies between the two studies indicate that further research is needed to better understand the relationship between spider food and the stability of their gut microbiota.

Variations in both nutritional level and the microbial community associated with their prey can both influence predators’ gut microbiota [41]. We collected two species of ants from different geographic sites to research food-induced gut bacterial community changes. Previous studies suggested that feeding fish with certain species improves muscle function [36]. The protein content in diets varies considerably, even in the same species [42]. To achieve a more comprehensive measurement of dietary differences, we evaluated compositional variations in protein, GT, trehalose, and glucose levels, three of which varied significantly between the *L. niger* and *T. caespitum* ant diets. In a previous study of ants, the authors found similarities in the bacterial communities between species of the same trophic level [43]. Conversely, some studies have provided evidence that the bacteria community is significantly varied across species rather than biogeography though their dominant bacteria are *Bacteroides, Helicobacter, Robinsoniella Polyrhachis* [35,44]. To determine whether the microorganisms in spiders’ food impact their gut microbiota, we analyzed the microbial diversity of feed ants dividing into three groups. We found that the microbiota composition of the two kinds of feed ants varied significantly, despite all belonging to Formicidae. As far as we are acknowledged, no studies have been reported about the microbiome of the *T. caespitum* ants. Díez-Méndez et al. found that Actinobacteria were the most abundant (*Streptomyces*, *Brevibacterium*, *Micrococcus*, *Rhodococcus*) bacteria, followed by Firmicutes (*Bacillus*, *Paenibacillus*, *Staphylococcus*), in the midgut of *L. niger* [45]. The reasons behind the difference in bacterial composition between the study and our results remains to be determined.

We used Illumina MiSeq sequencing of 16S rRNA genes to test whether spiders that ate different ants would also exhibit a difference in microbial composition. Our results demonstrated that the gut microbiota in all spiders maintained a consistent composition, with *Pseudomonas* as the dominant genus, which is known for its metabolic diversity and its ability to colonize many niches, including the gut [46]. To ensure the abundance of *Pseudomonas* did not result from contamination, we performed negative extraction and PCR control, which was visualized using a 2% agarose gel. Our results found no band for the negative extraction and PCR control and an obvious band for the positive control. The presence of this OTU makes it likely to be unintentionally sequenced; however, this OTU accounted for more than 90% of total sequences in our experimental samples. Our results indicate that the high percentage of *Pseudomonas* in our study was not due to contamination. Compared with common spiders living in cotton fields (where 237 genera were detected at the genus level [26]), *C. campanulata* could maintain a relatively simple gut environment. This could be due to its limited diet. Intriguingly, *Badumna Longinqua*, whose diet includes crickets and fruit flies, showed the opposite result [47]. Its gut microbiome experiences pronounced temporal fluctuations and slowly approached its initial state after more than 658 h. The difference between the results obtained by our study and that of previous research could be due to several factors, including the different spider species used and their rearing conditions. Their prey typically contains a rich microbiome, and the spiders sometimes ingest and maintain certain microbes from prey in their gut. This mimics a pattern of stability in the spider’s gut microbiota. Additionally, spider habitat and taxonomy could influence their gut microbiota [48], however, without knowing the natural state of the spider microbiomes obtained from the field, we can only conclude that the gut bacterial communities in groups fed different diets were similar, despite differences in their nutrients or ingested microbes.

Comparing the composition of the microbial community between predators and prey at the intraspecific level, we found that *C. campanulata* could maintain a relatively high level of *Pseudomonas*, which has been reported to produce tannase to detoxify or tolerate tannins in plant-based diets [49,50], but whether the function is relevant for spiders or the bacterium possesses another function in the spider’s gut needs further studies to verify. Interestingly, we detected no *Acinetobacter* in the spiders, despite its prevalence in the feed ants. This implies that not all microorganisms obtained from food can successfully colonize a spider gut, suggesting the host can prevent the colonization of new bacteria, possibly as a way to defend against pathogens [51]. In most cases, the intestinal bacterial community is short-lived and opportunistic, with widely diverse bacteria stemming from a constantly changing environment [52,53,54]. This stabilization of gut microbial diversity could be due to microbial competition or interference following the introduction of exogenous bacteria [55]; however, further investigation is required to confirm either of these hypotheses.

## 5. Conclusions

Totally, we isolated 682 genera from 356 families in the ants and 456 genera from 258 families in the spiders. The dominant bacteria in the ants were *Pseudomonas* spp., *Enterobacteriaceae* spp., *Acinetobacter* spp., and *Paraburkholderia* spp. Moreover, the ants in the LN_WH group have abundant bacteria in *Staphylococcus* and *Novosphingobium* genera. As for spiders, their guts are dominated by *Pseudomonas* spp. and *Enterobacteriaceae* spp. When we analyzed the microbial composition in three spider groups with different diets, we observed no dynamic changes in gut microbiota. This indicates that diet has minimal effect on the gut microbiota of *C. campanulata*, despite variations in feed microbial community and nutrients, ensuring normal physiological function and development. It is worth noting that identifying a mechanism by which this action takes place was beyond the scope of this study. We cannot estimate the impact of microbiota from the soil environment, which should be evaluated. Future studies should include additional types of prey and spiders to analyze these results on a larger scale and outline a potential mechanism for host regulation in gut microbial homeostasis and pay more attention to the microbiota from the environment. The analysis of spiders’ intestinal microbiota in conjunction with soil layer microorganisms analysis is also a worthwhile endeavor to determine if the dominant microorganisms in the spiders’ gut microbiota are a result of their environment. This work provides a blueprint for future investigation into the gut microorganisms of spiders and highlights the potential existence of a unique gut modulatory mechanism.

## Figures and Tables

**Table 1:** Richness and diversity estimation (mean ± SEM) of the bacterial community in the gut of six groups.

Sample	Species Richness Indices		Species Diversity Indices	
	ACE	Chao1	Shannon Diversity	Simpson Diversity
LN_WH	275.37 ± 60.72	238.20 ± 37.70	0.45 ± 0.08 ^b^	0.84 ± 0.02 ^a^
TC_WH	195.98 ± 78.32	198.23 ± 78.20	0.80 ± 0.24 ^b^	0.77 ± 0.06 ^a^
TC_LY	280.55 ± 97.26	278.61 ± 39.20	2.10 ± 0.72 ^a^	0.41 ± 0.13 ^b^
CC-LN_WH	331.10 ± 62.28 ^a^	206.76 ± 30.74	0.39 ± 0.07	0.87 ± 0.02
CC-TC_WH	152.03 ± 31.98 ^b^	114.44 ± 23.18	0.55 ± 0.28	0.80 ± 0.10
CC-TC_LY	268.50 ± 53.62 ^ab^	233.65 ± 60.98	0.48 ± 0.06	0.84 ± 0.01

Letters indicate significant differences across regimens (*p* < 0.05).

## Data Availability

All data used in this study are available from the NCBI SRA under the BioProjects ID: PRJNA742878 (https://www.ncbi.nlm.nih.gov/sra/PRJNA742878, accessed on 27 July 2021).

## Supplementary Materials

The following are available online at https://www.mdpi.com/article/10.3390/microorganisms9112358/s1, Figure S1. The content of protein (a), trehalose (b), TG (c), and glucose (d) in *Tetramorium caespitum* of different locations. TC_LY, *T. caespitum* Luoyang; TC_WH, *T. caespitum* Wuhan; Asterisks indicate statistically significant differences (* *p* < 0.05; *** *p* < 0.001); NS, not significant. Figure S2. Rarefaction curves of OTUs for the bacterial communities from ants and spiders. The rarefaction curves of determined tags tend to approach the saturation plateau. TC_LY, *T. caespitum* Luoyang; TC_WH, *T. caespitum* Wuhan; LN_WH, *L. niger* Wuhan; CC-, spiders fed different diets. Figure S3. Rank-Abundance curves to evaluate species diversity at the OTU level in ants (a) and spiders (b). The abscissa represents the ordinal of the OTU, and the ordinate represents the abundance of the OTU. TC_LY, *T. caespitum* Luoyang; TC_WH, *T. caespitum* Wuhan; LN_WH, *L. niger* Wuhan; CC-, spiders fed different diets. Table S1. The number of species at the genus level in each sample. TC_LY, *T. caespitum* Luoyang; TC_WH, *T. caespitum* Wuhan; LN_WH, *L. niger* Wuhan; CC-, spiders fed different diets. Table S2. Taxonomic composition of bacterial community at the genus level in each sample (>1%). The abundance is presented in terms of percentages of the total sequences in a sample. Enterobacteriaceae (family) was used for the unclassified group at the genus. TC_LY, *T. caespitum* Luoyang; TC_WH, *T. caespitum* Wuhan; LN_WH, *L. niger* Wuhan; CC-, spiders fed different diets. Table S3. Principal coordinate analysis (PCoA) of unweighted UniFrac analysis demonstrated that there was significant variation among ants (PERMANOVAR, *p* < 0.05). TC_LY, *T. caespitum* Luoyang; TC_WH, *T. caespitum* Wuhan; LN_WH, *L. niger* Wuhan; CC-, spiders fed different diets.
