# Supplementary figures and images for: Differing Dietary Nutrients and Diet-Associated Bacteria Has Limited Impact on Spider Gut Microbiota Composition
Source: Microorganisms. 2021 Nov 15;9(11):2358. doi: 10.3390/microorganisms9112358 (PMC8618231; doi:10.3390/microorganisms9112358)

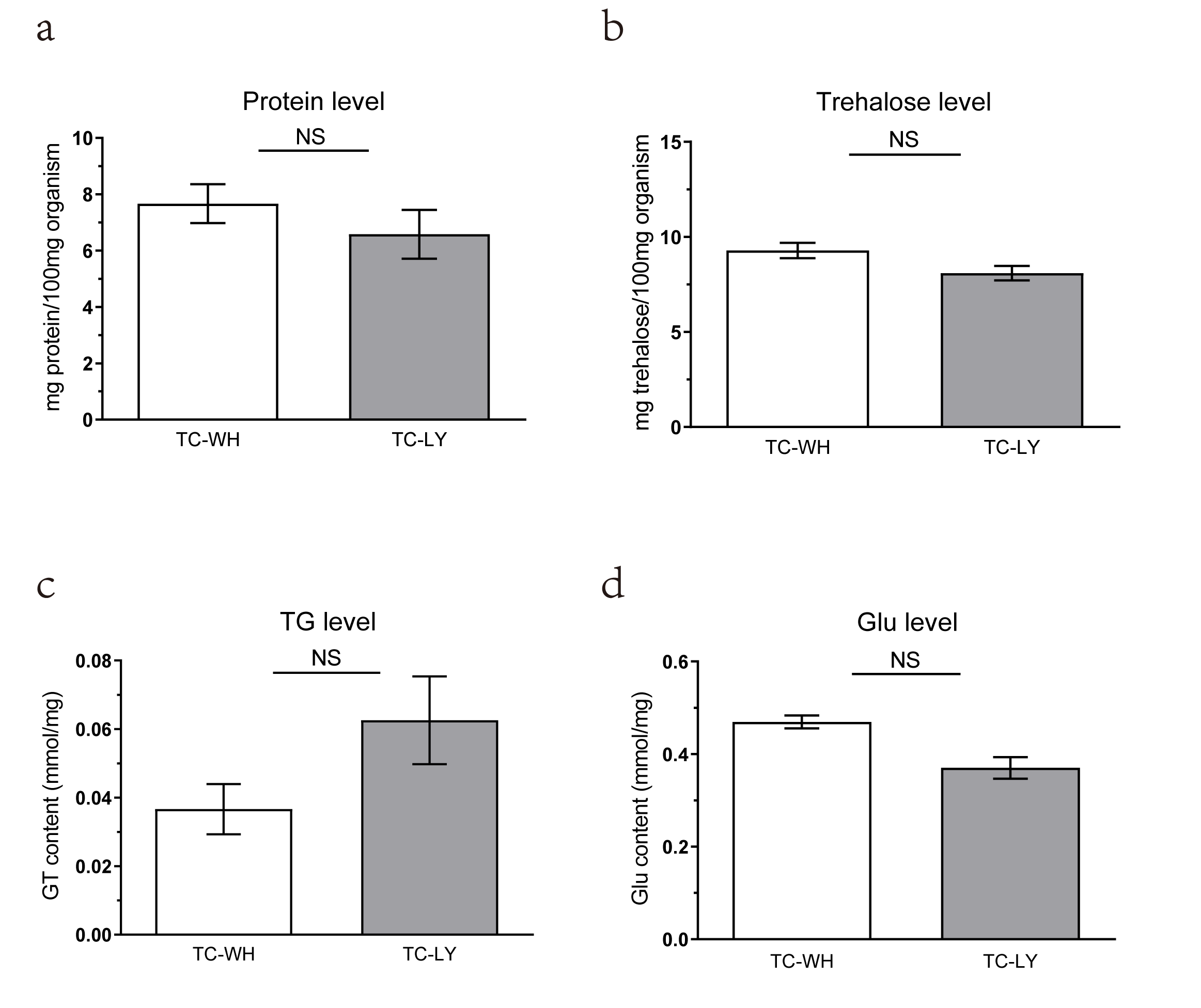

Supplement: Supplementary file 1 [file microorganisms-09-02358-s001.zip › microorganisms-1452291-supplementary-1/Supplementary Figure 1.tif]

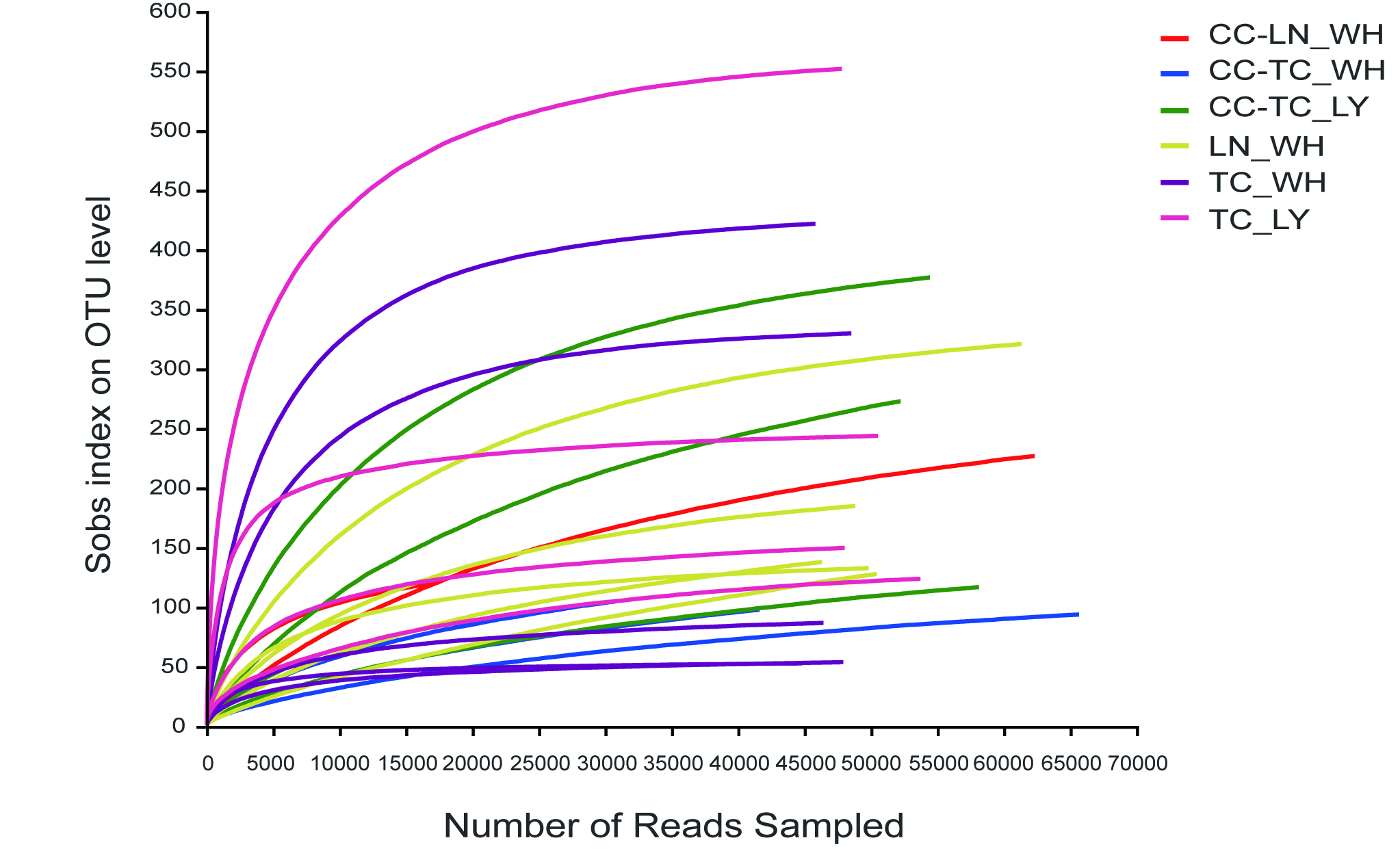

Supplement: Supplementary file 1 [file microorganisms-09-02358-s001.zip › microorganisms-1452291-supplementary-1/Supplementary Figure 2.tif]

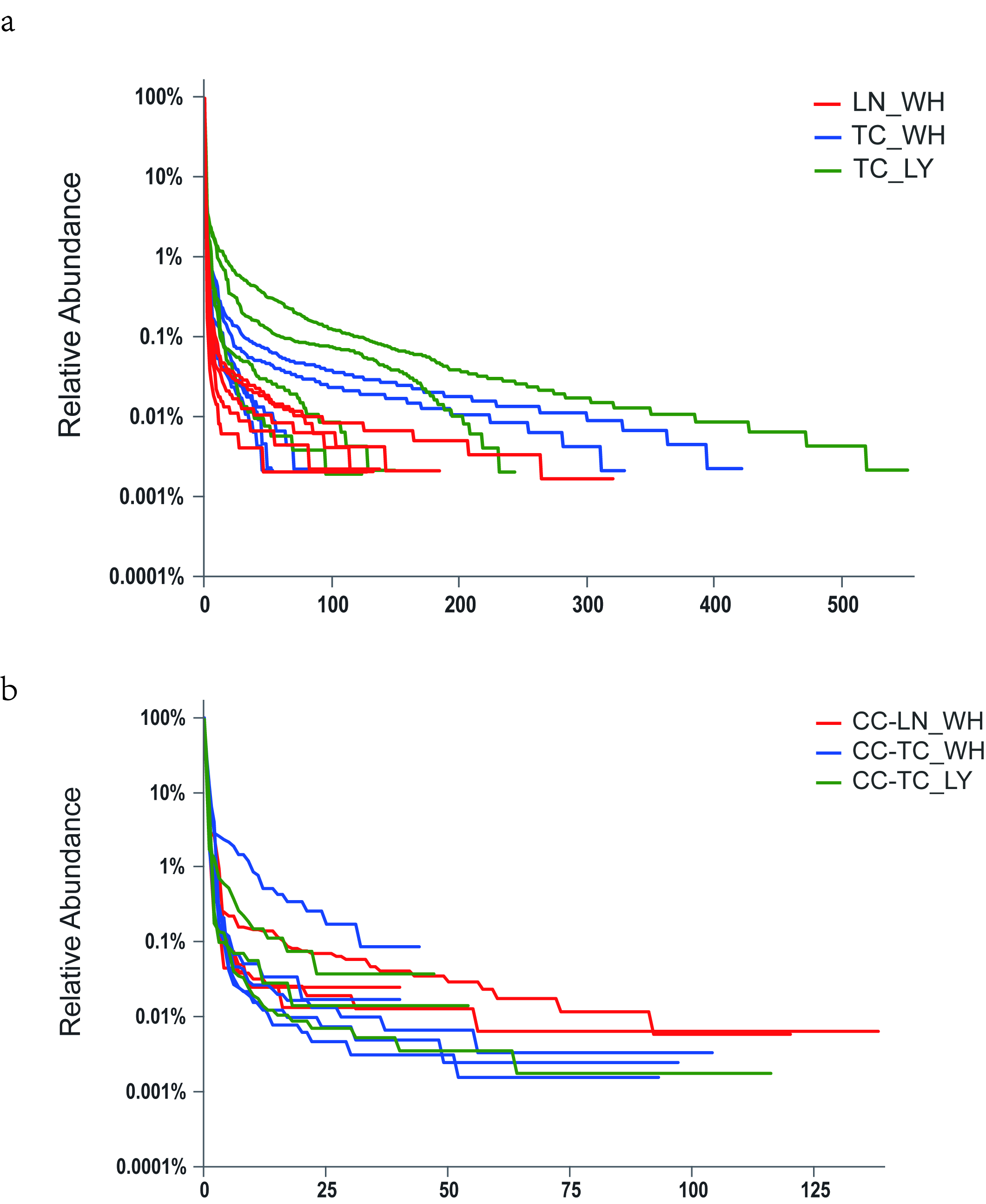

Supplement: Supplementary file 1 [file microorganisms-09-02358-s001.zip › microorganisms-1452291-supplementary-1/Supplementary Figure 3.tif]
